# Supplementary figures and images for: Biosynthetic proteins targeting the SARS-CoV-2 spike as anti-virals
Source: PLoS Pathog. 2022 Sep 6;18(9):e1010799. doi: 10.1371/journal.ppat.1010799 (PMC9481167; doi:10.1371/journal.ppat.1010799)

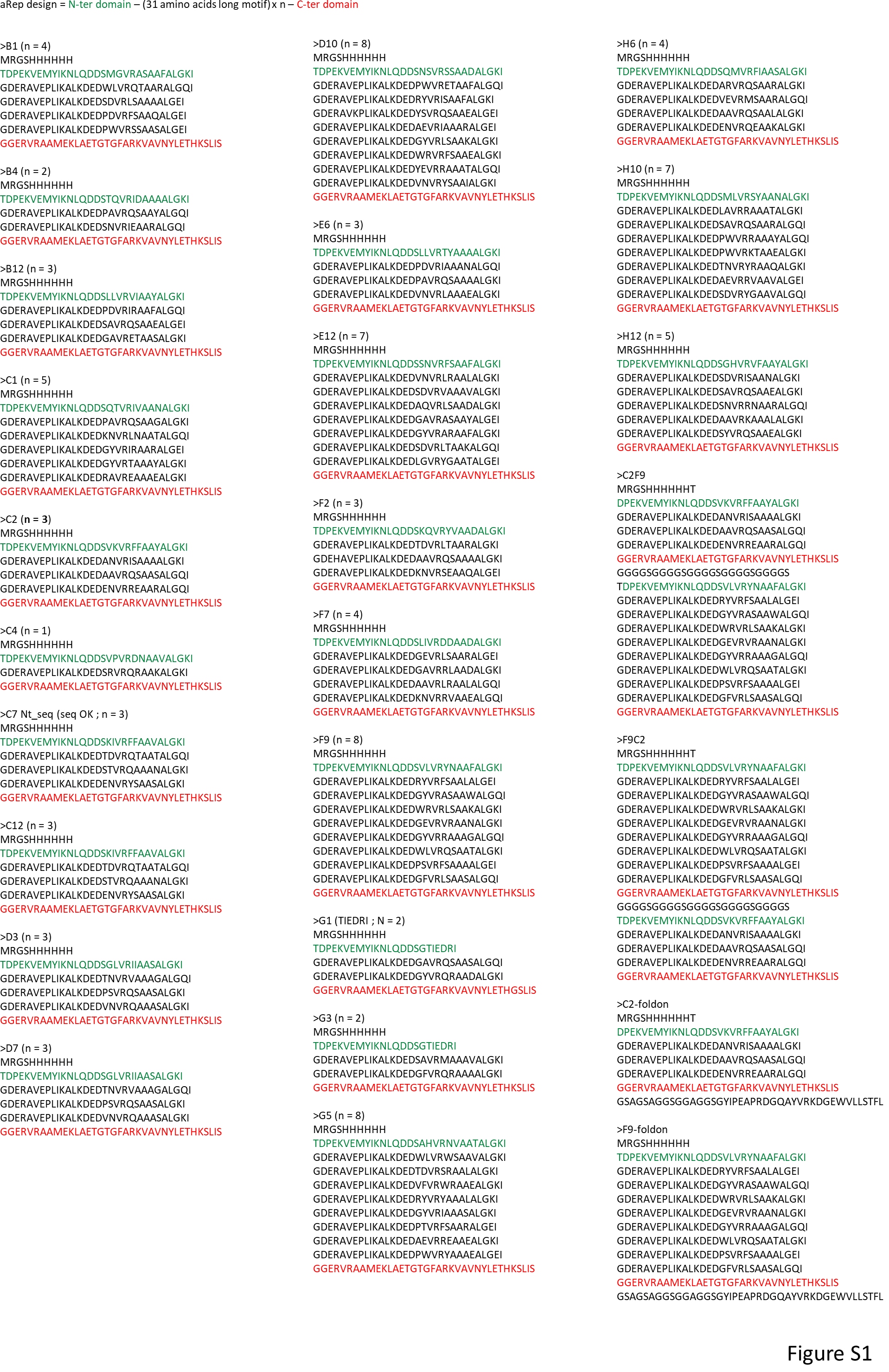

Supplement: S1 Fig — (TIF) [file ppat.1010799.s001.TIF]

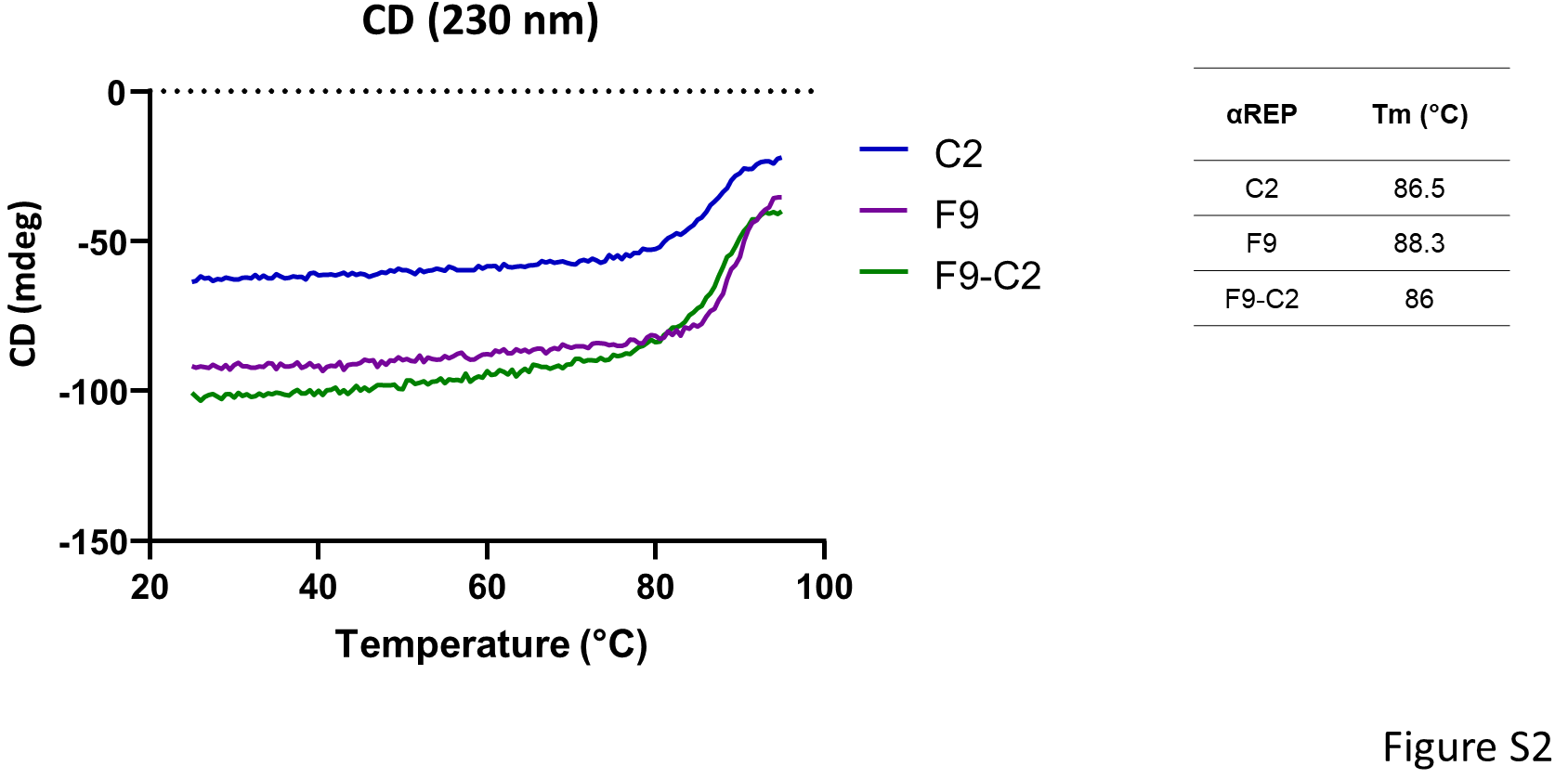

Supplement: S2 Fig — (TIF) [file ppat.1010799.s002.TIF]

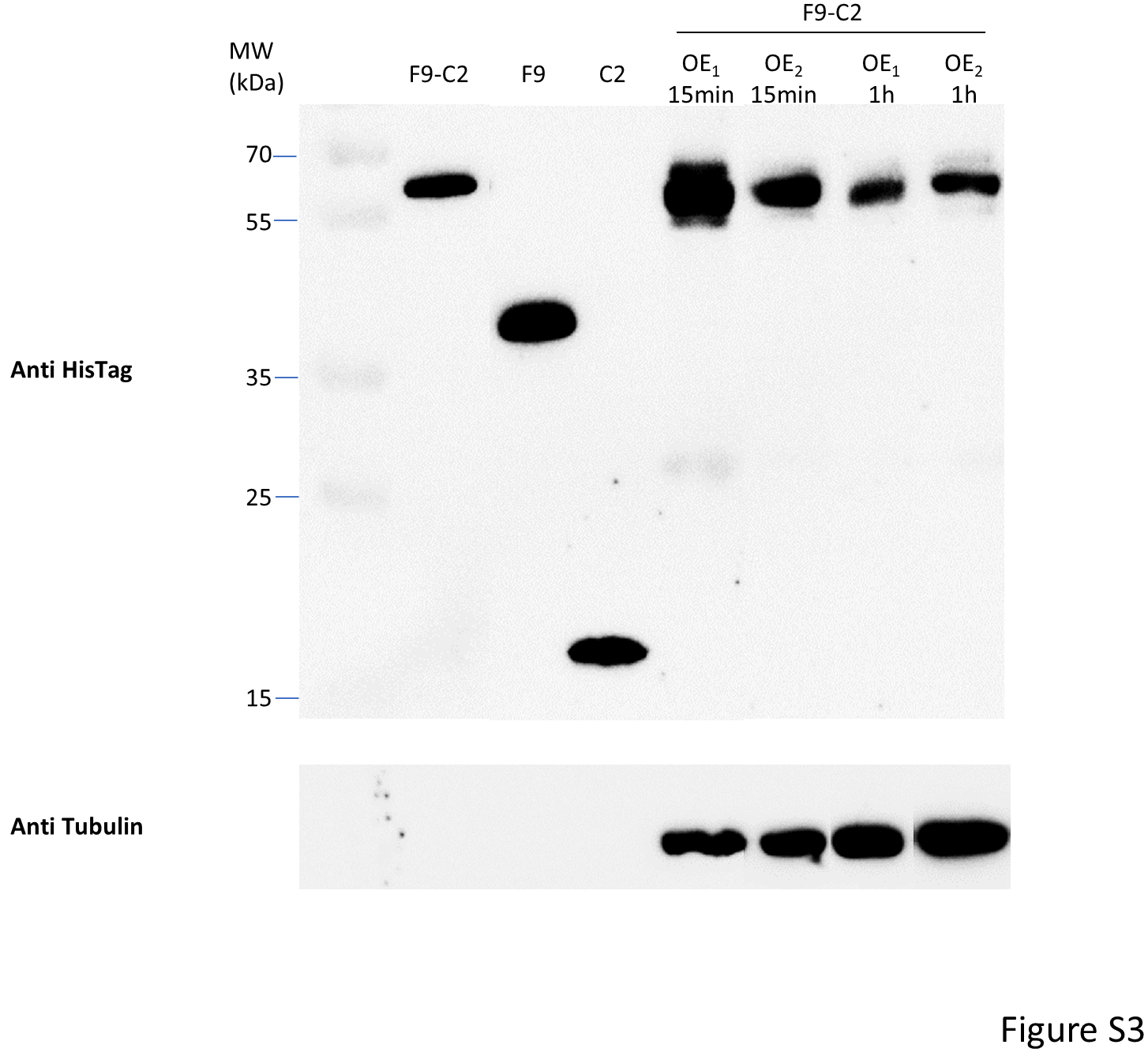

Supplement: S3 Fig — His-tagged αReps are revealed with an anti-HisTag mouse antibody and with a secondary HRP-anti-mouse antibody using a horseradish peroxidase substrate. OE: Olfactory epithelium. His-tagged F9-C2, F9 and C2 (500 ng) were loaded as internal controls. (TIF) [file ppat.1010799.s003.TIF]

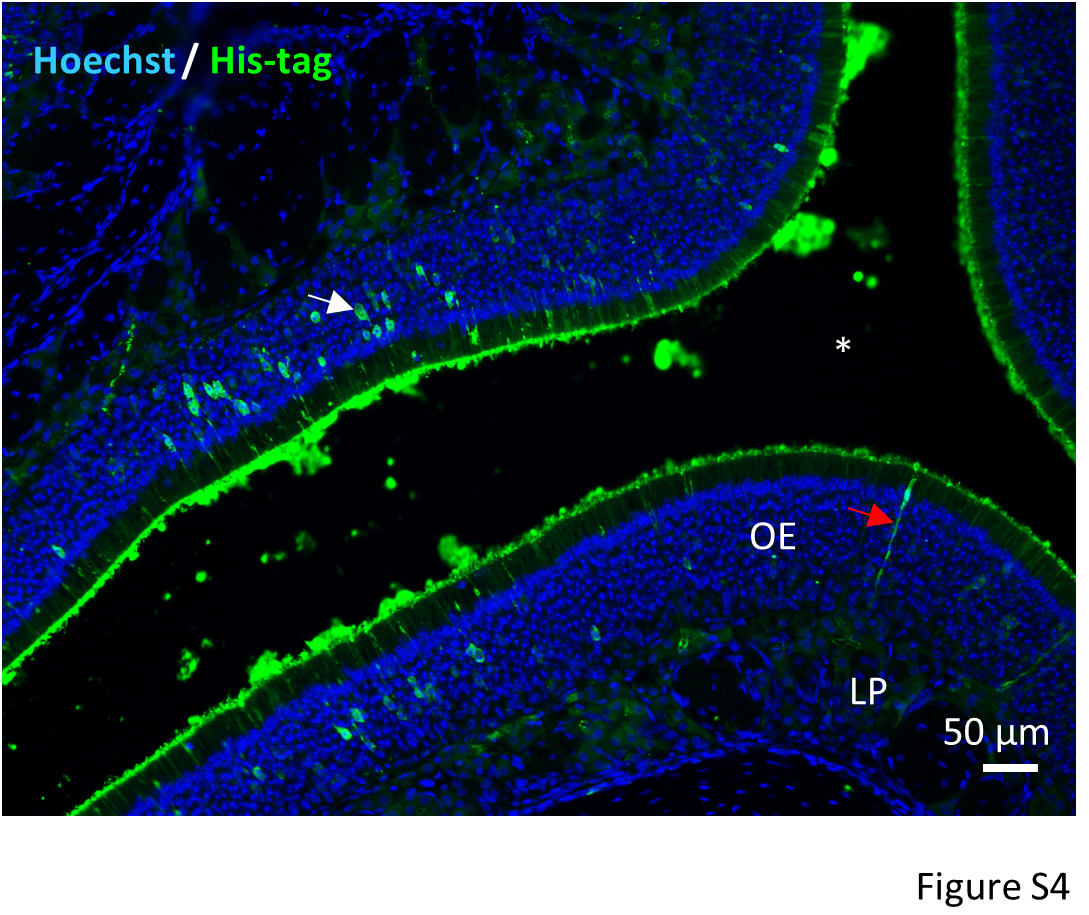

Supplement: S4 Fig — F9-C2 is mainly present in the mucus layer but some cells have integrated them (OE: Olfactory Epithelium / LP: Lamina Propria / white asterisk Lumen of the nasal cavity / red arrow: sustentacular cell like shape / white arrow (olfactory sensory neuron like shape). (TIF) [file ppat.1010799.s004.TIF]

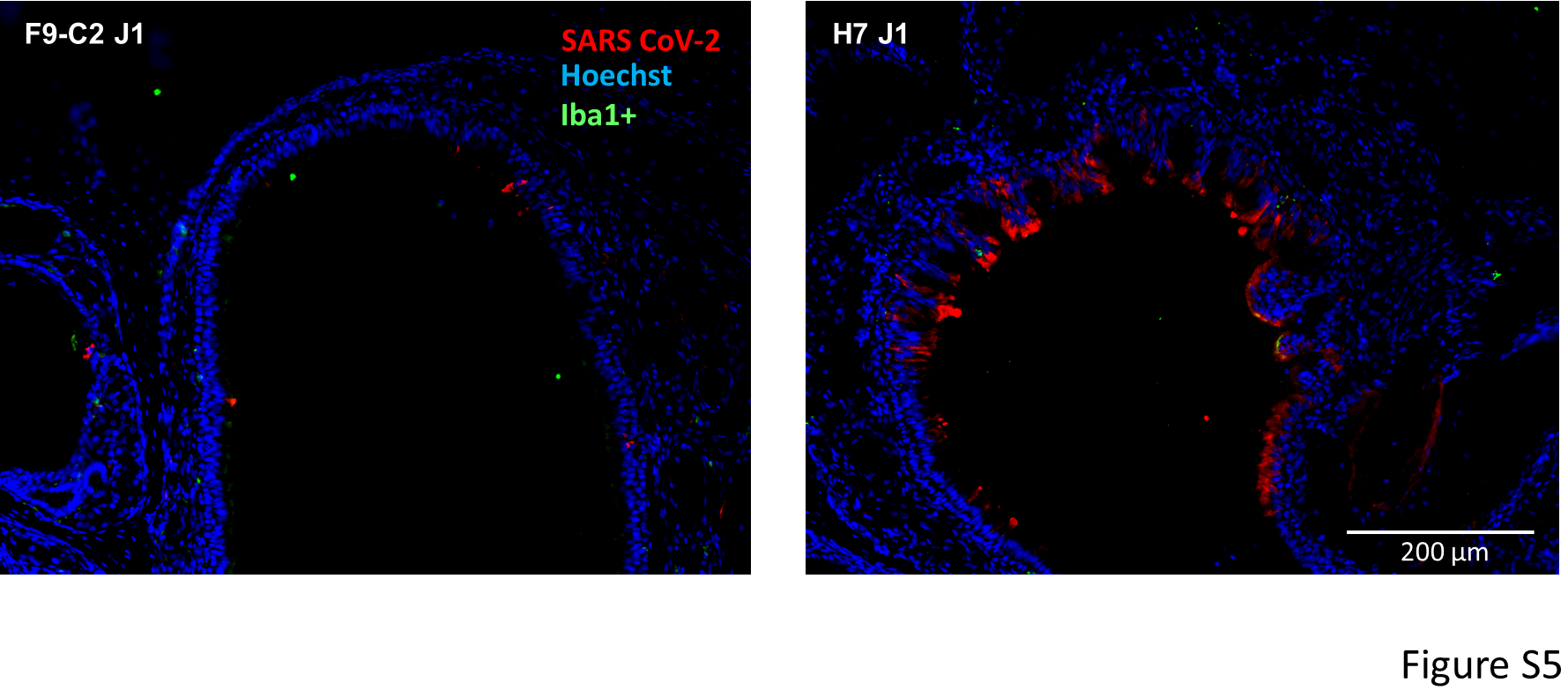

Supplement: S5 Fig — SARS-CoV-2 infected cells were revealed with an anti-N antibody. F9-C2 was found to protect the nasal cavity epithelium. (TIF) [file ppat.1010799.s005.TIF]
